# 

**Published:** 1898-03

**Authors:** 


TABLE OF CONTENTS ON LAST ADVERTISING PAGE.

Vol. XIII. MARCH, 1898. No. 9.

### TEXAS Medical Journal.

Subscription, $2.00 a Year, in Advance.
Single Copies, 25 Cents.

PUBLISHED AT AUSTIN, TEXAS, BY
F. E. DANIEL, M. D., & S. E. HUDSON, M. D.,
Editors and Proprietors.

Eastern Representative: Messrs. Monihan & Fairchild, St. Paul Building, 220 Broadway, New York City.

Entered at the Postoffice at Austin, Texas, as Second-class Mail Matter.

